# Draft Genome Sequence of Pseudomonas saudiphocaensis Strain AGROB56, Isolated from a Dairy Farm in New Zealand

**DOI:** 10.1128/MRA.01258-20

**Published:** 2021-01-07

**Authors:** Tanushree B. Gupta, Alexis N. Risson, Ruy Jauregui, Paul Maclean, Gale Brightwell

**Affiliations:** a Food Assurance Team, Hopkirk Research Institute, AgResearch, Palmerston North, New Zealand; b Knowledge and Analytics, Grasslands Research Centre, AgResearch, Palmerston North, New Zealand; Loyola University Chicago

## Abstract

Here, we report the draft genome sequence of a new Pseudomonas saudiphocaensis strain, AGROB56, with lipolytic potential, isolated from a sheep dairy farm in New Zealand. The genome is 3.61 Mbp, with a GC content of 61.1%, and the genome sequence was found closely related to *Pseudomonas saudiphocaensis* 20 BN^T^.

## Abstract (precis)

Here, we report the draft genome sequence of a new Pseudomonas saudiphocaensis strain, AGROB56, with lipolytic potential, isolated from a sheep dairy farm in New Zealand. The genome is 3.61 Mbp, with a GC content of 61.1%, and the genome sequence was found closely related to *Pseudomonas saudiphocaensis* 20 BN^T^

## ANNOUNCEMENT

*Pseudomonas* species are ubiquitously found in environments, from soil to humans and plants (1–5). This genus was first proposed in 1894 and so far consists of around 254 species (6). They are Gram-negative bacteria, usually aerobic, but can be facultative anaerobes (7–9). While some *Pseudomonas* species can be associated with pathogenicity, some species have been applied as bioremediation agents (5, 10–14), and some are known for their dairy spoilage potential, such as Pseudomonas fragi, P. lundensis, and P. fluorescens (15, 16).

A new species of *Pseudomonas*, *P. saudiphocaensis* (type strain 20 BN), was isolated in 2012 from air samples in the city environment of Makkah, Saudi Arabia. As yet, limited information is available on this strain, including any beneficial or pathogenic traits (17).

Here, we report the whole-genome sequence of a new *Pseudomonas saudiphocaensis* strain, AGROB56, that was isolated from woodchip bedding samples from a sheep dairy farm in North Island of New Zealand. The new strain showed lipolytic activity, indicating it to be a food spoilage bacterium. The sequences obtained will be used to compare genomic data of the new strain to those of the type strain available.

Samples were processed using the methodology, with slight modifications, described previously by Gupta and Brightwell (18). Briefly, woodchip bedding material (25 g) was weighed in a stomacher bag, suspended in 100 ml of phosphate buffer (PB), and homogenized well using a stomacher. The suspension was centrifuged at 3,466 × *g* for 1 h, and the pellet was resuspended in 25 ml of PB. One milliliter of the suspension was serially diluted, plated onto cetrimide-fucidin-cephalosporin (CFC) agar plates, and incubated at 25°C (Fort Richard, New Zealand) to isolate *Pseudomonas* strain AGROB56 (19). Lipolytic activity was preliminarily investigated by visualizing a fluorescent halo, under UV light, around the colony grown for 48 h at 25°C on rhodamine B plates (20). The new strain was found to be lipolytic, indicating a potential food/dairy spoilage bacterium. Genomic DNA from the pure culture grown in tryptic soy broth at 25°C was extracted using the phenol-chloroform extraction method (21). Quality and concentration of DNA were determined using a Qubit 2.0 fluorometer (Thermo Fisher Scientific, USA).

The whole genome of *Pseudomonas* species strain AGROB56 was prepared via the NuGEN Celero DNA enzymatic library and sequenced using the Illumina MiSeq sequencing platform version 3 (Massey Genome Services, Palmerston North, New Zealand) to produce 628,743 paired-end reads of 301 base pairs, giving a coverage of 104-fold. The reads were quality trimmed, filtered, and assembled via A5-miseq pipeline version 20160825 with default settings (22). The assembly produced 20 contigs with a total genome size of 3.6 Mb, an *N*_50_ value of 481 kb, and a GC content of 61.1%. A BUSCO version 3.0.2 (23) test using the bacterial reference produced a completeness score of 99.3%.

Comparative genomic analysis was performed with the genome sequences of the new strain and *Pseudomonas saudiphocaensis* 20 BN^T^ using *in silico* DNA-DNA hybridization (dDDH) via the type (strain) genome server (TYGS; https://tygs.dsmz.de/) (24). A dDDH (d_6_) value of 92.8% was obtained, indicating the same species but with probable differences at strain level. Further studies are required to investigate these differences. A two-way average nucleotide identity test (http://enve-omics.ce.gatech.edu/ani/) was carried out as well, producing a 98% value matching with *Pseudomonas saudiphocaensis* 20 BN^T^ (25).

As part of the submission process, NCBI annotated the genomic scaffolds with PGAP version 4.13 (26), resulting in 3,396 genes being annotated in total.

### Data availability.

This whole-genome shotgun project has been deposited at DDBJ/ENA/GenBank under the accession number JADDOO000000000. The version described in this paper is the first version, JADDOO010000000. The raw sequencing data have been deposited at the SRA under BioProject accession number PRJNA670249.
